# Study on Properties of Additive Manufacturing Ta10W Alloy Laser-Welded Joints

**DOI:** 10.3390/ma17246268

**Published:** 2024-12-22

**Authors:** Rui Zhen, Liqun Li, Yunhao Gong, Jianfeng Gong, Yichen Huang, Shuai Chang

**Affiliations:** 1 State Key Laboratory of Precision Welding & Joining of Materials and Structures, Harbin Institute of Technology, 92 West Dazhi Street, Harbin 15001, China; 2 Zhengzhou Research Institute, Harbin Institute of Technology, Zhengzhou 450000, China

**Keywords:** SLM, Ta10W alloy, laser welding, anisotropy, mechanical properties

## Abstract

This investigation focuses on Selective Laser Melting (SLM)-fabricated thin-walled Ta10W alloy components. Given the inherent limitations of SLM in producing large-scale, complex components in a single operation, laser welding was investigated as a viable secondary processing method for component integration. The study addresses the critical issue of weldability in additively manufactured tantalum-tungsten alloys, which frequently exhibit internal defects due to process imperfections. Comprehensive analyses were conducted on weldability, microstructural evolution, texture intensity, and mechanical properties for welds oriented along both traveling and building directions. Results demonstrate that welds oriented along the traveling direction exhibit superior performance characteristics, including enhanced tensile strength, increased yield strength, improved elongation, and reduced texture intensity compared to building direction welds. Notably, grain orientation alignment between the weld zone and base material was observed consistently in both directional configurations.

## 1. Introduction

The Ta10W alloy represents an advanced high-temperature refractory material system, comprising a strategic combination of tantalum and tungsten. This alloy exhibits exceptional mechanical properties, characterized by superior strength retention, notable plastic deformation capability, excellent corrosion resistance, and favorable weldability under elevated temperature conditions. These characteristics, particularly its ultra-high temperature performance capabilities, make the Ta10W alloy an optimal candidate for advanced aerospace structural applications [1].

Welding technology constitutes a fundamental manufacturing process in tantalum-tungsten alloy fabrication, playing a crucial role in complex structure assembly and high-performance component integration. In aerospace applications, electron beam welding of Ta10W alloys is extensively utilized for manufacturing high-temperature components, specifically rocket engine nozzles and thermal protection systems. Laser welding technology demonstrates particular efficacy in the production of small-scale, precision components, especially for advanced aerospace microsystems. In nuclear applications, Ta10W alloy welding is instrumental in fabricating reactor components and radiation-resistant fuel cladding, exhibiting remarkable stability under conditions of elevated temperature and neutron irradiation. Furthermore, friction stir welding (FSW), characterized by its low-temperature processing capabilities, has found widespread implementation in chemical industry applications, particularly in the fabrication of Ta10W alloy-lined corrosion-resistant vessels and pipeline systems. The emergence of additive manufacturing technologies, coupled with integrated welding methodologies, has significantly enhanced the manufacturing efficiency and performance optimization capabilities for geometrically complex Ta10W alloy components [2,3].

The current state of laser additive manufacturing for Ta10W alloys exhibits certain technological limitations, manifesting in the formation of internal defects that significantly impact the mechanical integrity of fabricated components. This technological constraint represents a significant challenge in additive manufacturing applications. Additionally, conventional thermomechanical processing methods demonstrate inadequacies in achieving optimal weldability, strength, and ductility characteristics. Gryguć et al. [4,5] conducted comprehensive investigations on the fatigue behavior of closed-die forged AZ80 magnesium alloy under multiaxial loading conditions. Their findings revealed that disproportionate loading significantly compromises fatigue life, inducing a transition from shear-dominated to mixed-mode cracking mechanisms. Furthermore, their research demonstrated that crack morphology is substantially influenced by the initial material condition and process-induced microvoids introduced during forging operations. These investigations emphasize the critical role of matrix defects in determining crack orientation and fatigue life characteristics, particularly under conditions where shear strain energy density predominates.

S. Seyyedin et al. [6] employed plasma sintering subsequent to mechanical alloying to systematically investigate the correlation between alloying duration and the resultant microstructural and mechanical properties of Ta-W alloys. Their research demonstrated that extended alloying periods facilitate enhanced grain refinement, leading to improved strength and elongation characteristics. P.N. Browning et al. [7] corroborated these findings through spark plasma sintering investigations, while Guo et al. [8] successfully demonstrated the feasibility of additively manufacturing thin-walled Ta-W alloy plates. While conventional manufacturing methodologies exhibit limitations in producing high-precision components, additive manufacturing technologies offer distinct advantages in the net-shape fabrication of small-scale, precision components, accompanied by significant economic benefits.

The powder bed-based selective laser melting technology, while offering precise control over component fabrication, inherently imposes dimensional constraints on structural components. For large-scale tantalum-tungsten alloy structures, a hybrid manufacturing approach combining selective laser melting and laser welding technologies presents a viable solution. Consequently, establishing reliable joining methodologies between additively manufactured components becomes paramount [9]. Chen et al. [10] successfully achieved electron beam welding of TC4 alloy with Ta-W, attaining tensile strength values of 714 MPa. Xu et al. [11] implemented argon arc welding techniques for high-tungsten-content Ta alloys, achieving tensile strengths ranging from 438 MPa to 573 MPa, contingent upon the specific welding methodology employed. Laser welding technology, characterized by minimized heat input and superior depth-to-width ratio characteristics, demonstrates particular advantages in the fabrication of precision components with complex geometries [12].

This investigation focuses specifically on the implementation of laser welding in the context of additively manufactured Ta10W alloy, with particular emphasis on the systematic characterization of microstructural evolution and mechanical property variations across different welding orientations.

## 2. Materials and Methods

The base material utilized in this investigation comprises additively manufactured tantalum-tungsten alloy with the following precise chemical composition (wt.%): 9.67 W, 0.01 Ti, 0.01 Nb, 0.01 Mo, 0.005 Si, 0.01 Ni, 0.005 C, and the remaining Ta.

The experimental apparatus employed for laser welding operations incorporates three primary subsystems: a laser source unit, a laser delivery head, and an integrated cooling system. The laser source consists of a JPT laser CW4000 system (JPT Opto-electronics Co., Ltd., Shenzhen, China), characterized by the following operational parameters: maximum output power of 4000 W, central wavelength of 1080 nm, power consumption ceiling of 14 kW, and maximum modulation frequency of 5000 Hz. This laser system exhibits several advantageous characteristics, including superior electro-optical conversion efficiency, minimal energy consumption, maintenance-free operation protocols, flexible fiber-optic beam delivery capabilities, and enhanced operational versatility, rendering it particularly suitable for industrial applications encompassing laser cutting, welding, and related processes. The thermal management system comprises a CWFL-4000 fiber laser chiller (Guangzhou Teyu Electromechanical Co., Ltd., Guangzhou, China), incorporating dual independent temperature control circuits for simultaneous cooling of both the laser source and delivery head, thereby optimizing both operational costs and spatial requirements. The mechanical manipulation system utilizes a KUKA robotic arm (KUKA Aktiengesellschaft Co., Ltd., Augsburg, Germany) assembly, mechanically integrated with the laser delivery head.

**Figure 1:**
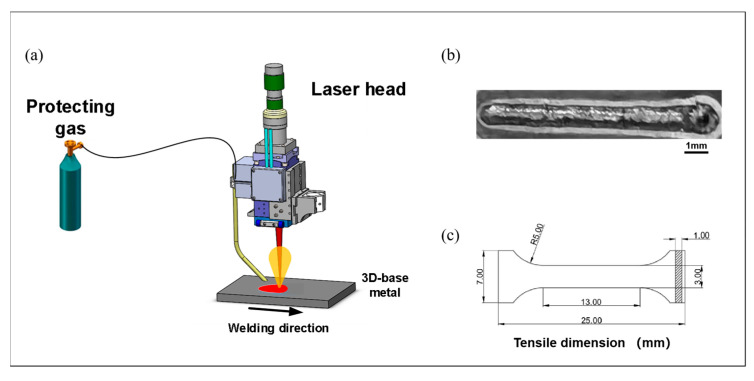
Welding equipment, weld morphology, and the diagram of tensile specimen size. (**a**) Welding assembly drawing. (**b**) Macro morphology of joints. (**c**) Schematic diagram of tensile specimen.

The additive Ta10W alloy base material specimens were precisely dimensioned to 43 mm × 25 mm × 2 mm, with weld morphology as illustrated in Figure 1b. Laser welding operations were conducted along both traveling and building directions. The experimental matrix encompassed five distinct welding parameter sets: laser power settings of 1500 W combined with welding velocities of 1.5 m/min, 1.2 m/min, and 1 m/min, supplemented by additional trials at 1800 W and 2000 W with constant welding velocity of 1 m/min. These parameters correspond to specific heat inputs of 60 J/mm, 75 J/mm, 90 J/mm, 108 J/mm, and 120 J/mm, respectively.

For metallographic examination, designated sections of the weld bead regions were extracted and subsequently prepared through either hot-mounting or cold-mounting procedures to facilitate comprehensive microstructural analysis. The metallographic preparation protocol involved sequential grinding operations utilizing progressively finer abrasive media, with grinding orientation alternated orthogonally between successive grades. Surface finishing was continued until mirror-like surface characteristics with no discernible scratch patterns were achieved, typically progressing through 5000 grit, followed by diamond suspension polishing procedures. Post-cleaning operations utilizing ethanol and subsequent drying were performed prior to chemical etching. The etching solution was precisely formulated using a 5:2:4 volumetric ratio of 98% concentrated sulfuric acid, 68% nitric acid, and 40% hydrofluoric acid. Given the volatile nature of hydrofluoric acid, the etching solution was prepared immediately before application, with a standardized etching duration of 30 seconds. Post-etching procedures included ultrasonic cleaning for 5 minutes followed by forced-air drying prior to microscopic examination. 

The microstructural characterization of the weld bead was conducted utilizing both high-magnification optical microscopy and scanning electron microscopy (SEM) techniques. Sample preparation protocols remained consistent with those established for macrostructural observation. A Keyence VHX-1000E super-depth-of-field optical microscope (Keyence Co., Ltd., Osaka, Japan) was employed for optical metallographic analysis, while a Zeiss Gemini560 field-emission (Carl Zeiss AG Co., Ltd., Oberkochen, Germany) SEM facilitated high-resolution electron micrography and energy-dispersive X-ray spectroscopy (EDS) for compositional analysis. For electron backscatter diffraction (EBSD) analysis, which necessitates superior surface quality, Ta10W alloy specimens underwent additional vibration polishing procedures, as conventional mechanical polishing proved insufficient, and electrochemical polishing demonstrated inadequate effectiveness due to the alloy’s unstable electrochemical behavior. The EBSD analysis provided comprehensive data regarding grain size distribution and crystallographic orientation characteristics.

Tensile test specimens were fabricated according to the dimensional specifications illustrated in Figure 1c, utilizing wire electrical discharge machining (WEDM). The specimens underwent subsequent surface preparation using coarse abrasive media to eliminate machining artifacts and ensure dimensional consistency across the gauge section, followed by mechanical testing using a calibrated universal testing machine.

## 3. Results and Discussions

### 3.1. Effect of Heat Input on Weld Formation

**Figure 2:**
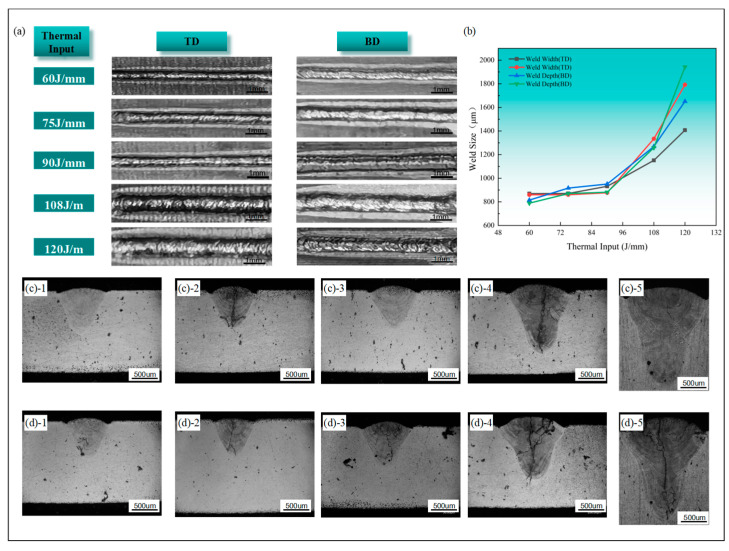
Welding results under different thermal inputs: (**a**) Surface morphology of the weld seam. (**b**) Relationship between weld dimensions and heat input. (**c**)-1 Weld in traveling direction at 60 J/mm; (**c**)-2 75 J/mm; (**c**)-3 90 J/mm; (**c**)-4 108 J/mm; (**c**)-5 120 J/mm. (**d**)-1 Weld in building direction at 60 J/mm; (**d**)-2 75 J/mm; (**d**)-3 90 J/mm; (**d**)-4 108 J/mm; (**d**)-5 120 J/mm.

Surface morphological characteristics of the weld under varying heat inputs at a constant defocus distance of + 1 mm are presented in Figure 2a. Systematic observation reveals a direct correlation between heat input magnitude and the resultant weld width. The quantitative relationship between weld dimensional parameters and heat input is illustrated in Figure 2b, demonstrating progressive increases in both weld width and depth with increasing heat input. Furthermore, the rate of dimensional expansion exhibits accelerated behavior at elevated heat input levels.

Metallographic analyses of welds produced under varying heat inputs and welding directions are presented in Figure 2c,d. Welds executed along the traveling direction exhibit significantly reduced incidence of internal crack formation and porosity defects compared to those produced along the building direction. This directional dependence can be attributed to the inherent anisotropic characteristics of the additively manufactured Ta10W alloy base material.

The cracks primarily consist of penetrating crystallization cracks. The additive Ta10W alloy base material tends to have numerous unfused defects, increasing the likelihood of crystallization cracks. Defects were observed within the weld beads in the building direction, regardless of the heat input. The 60 J/mm condition showed no significant defects for the welds in the traveling direction, while the 75 J/mm condition exhibited longitudinal cracks through the weld and two transverse cracks. The 90 J/mm weld had no cracks but showed honeycomb-like porosity at the reinforcement. The 108 J/mm weld had longitudinal cracks through the weld and several cracks at the grain boundaries, and further increasing the heat input to 120 J/mm resulted in a small number of cracks and pores, with crack lengths ranging from 67.57 µm to 304.06 µm and pore sizes from 29.39 µm to 76.62 µm. The observed porosity predominantly comprises process-induced defects attributable to keyhole instability during laser welding operations. According to liquid column stability principles, instability initiates when the liquid column length exceeds its perimetric dimensions, resulting in periodic necking and expansion phenomena at the upper region. As the heat source translates, surface tension effects induce the collapse of the necked and expanded regions of the liquid column, leading to detachment from the keyhole and subsequent bubble formation, which becomes entrapped within the weld seam. 

### 3.2. Microstructural Analysis

**Figure 3:**
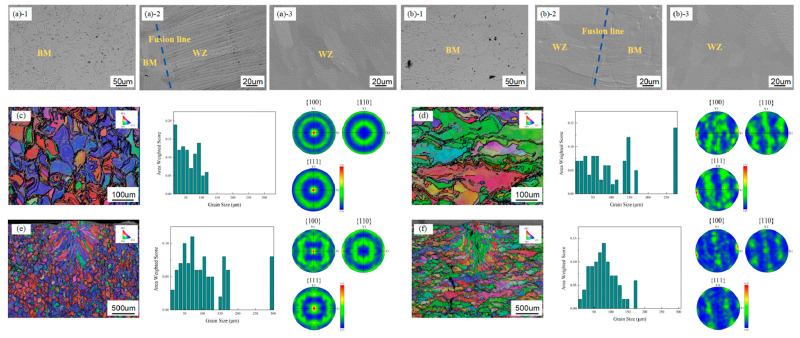
Metallographic diagram and EBSD results: (**a**) Building direction microstructural change. (**b**) Traveling direction microstructural change. (**c**) Building direction base metal EBSD results. (**d**) Traveling direction base metal EBSD results. (**e**) Welding zone of building direction EBSD results. (**f**) Welding zone of traveling direction EBSD results.

The microstructural characterization of the additively manufactured Ta10W alloy is presented in Figure 3a,b. The weld zone exhibits a predominantly bimodal dendritic structure with distinct directional orientations. Notably, the absence of a clearly delineated heat-affected zone (HAZ) can be attributed to the limited dimensional scale of the base material coupled with the high-velocity welding parameters employed.

Figure 3c,d illustrates the crystallographic orientation distribution within the base material. A significant directional variation in grain orientation is observed across the additive Ta10W alloy structure. In the building direction, the crystallographic texture is predominantly characterized by {001} (red) and {111} (blue) orientations, while the traveling direction exhibits a predominant {101} (green) orientation. The observed grain orientation patterns demonstrate pronounced preferential alignment, indicating a significant anisotropic behavior. The quantitative analysis of grain distribution reveals distinct variations in grain morphology: the building direction exhibits a relatively fine grain structure with mean equivalent circular diameter of 61.16 µm, while the traveling direction demonstrates coarser grain structure averaging 100.78 µm.

The crystallographic characteristics correlate with specific mechanical behavior patterns: {001} orientation typically exhibits enhanced ductility with reduced strength properties, while {101} and {111} orientations generally manifest elevated strength and hardness characteristics. Consequently, significant variations in tensile strength, yield strength, and ductility parameters are anticipated between building and traveling directions.

In accordance with the Hall–Petch relationship, the reduced grain dimensions typically correspond to enhanced yield strength and hardness properties, attributed to increased grain boundary density impeding dislocation motion. Thus, the building direction is expected to exhibit superior yield strength and hardness characteristics, albeit with potentially reduced ductility compared to the coarser-grained traveling direction.

The combined influence of textural variation and grain size differentiation contributes to the observed anisotropic mechanical behavior in the additively manufactured Ta10W alloy. The building direction, characterized by finer grain structure and pronounced texture, typically exhibits enhanced strength and hardness properties, while the traveling direction, with its coarser grain structure and distinct texture, demonstrates superior ductility and fatigue performance characteristics [13].

The laser selective melting process, characterized by layer-wise material deposition, generates maximum thermal gradients along the building direction, resulting in preferential grain growth and the formation of pronounced columnar structures [14]. These columnar grains undergo epitaxial growth mechanisms, whereby the previously solidified substrate experiences partial remelting during subsequent laser passes, facilitating continued grain growth along established crystallographic orientations. The inherently high melting point and thermal conductivity of Ta10W alloy subject the material to extreme thermal gradients and rapid solidification rates under concentrated heat sources such as laser or electron beams. These severe thermal conditions significantly influence grain growth directionality and microstructural orientation, typically manifesting in columnar grain formation parallel to heat flow vectors, resulting in pronounced anisotropic characteristics. In comparison to conventional additive manufacturing alloys, the elevated thermal conductivity of Ta10W promotes heat conduction-dominated thermal distribution patterns rather than heat accumulation, rendering grain orientation highly dependent on component geometry and thermal flow paths.

The high melting point of Ta10W results in an extensive solidification temperature range, leading to significant residual stress accumulation. The distribution of these residual stresses typically exhibits directional characteristics, further amplifying material anisotropy. Additionally, the combination of high thermal conductivity and rapid cooling rates may facilitate microcrack or hot crack formation, particularly in geometrically complex components, with these defects demonstrating preferential propagation along specific crystallographic planes compared to conventional additive manufacturing alloys.

The quantitative analysis reveals an average grain size of 75.44 µm in the traveling direction weld zone, with predominant {101} crystallographic orientation. Conversely, the building direction weld zone exhibits an average grain size of 87.57 µm, characterized by dominant {111} and {001} orientations. Grain boundary regions typically demonstrate superior strength compared to intragranular regions. The increased proportion of grain boundary area in finer-grained structures provides enhanced impediment to dislocation motion. The crystallographic misorientation between adjacent grains generates mutual constraint effects; finer grain structures result in individual grains being surrounded by a greater number of differently oriented neighbors, intensifying the mutual constraint phenomenon. The increased effective grain boundary area contributes to metal strengthening mechanisms. Furthermore, finer grain structures facilitate a more uniform distribution of deformation across multiple grains, mitigating localized stress concentration and thereby reducing susceptibility to deformation and crack initiation, ultimately enhancing metal plasticity and toughness. Observational evidence indicates that the finer grain structure in the traveling direction weld zone demonstrates appreciable suppression of crystallization crack formation.

### 3.3. Mechanical Properties Analysis

**Figure 4:**
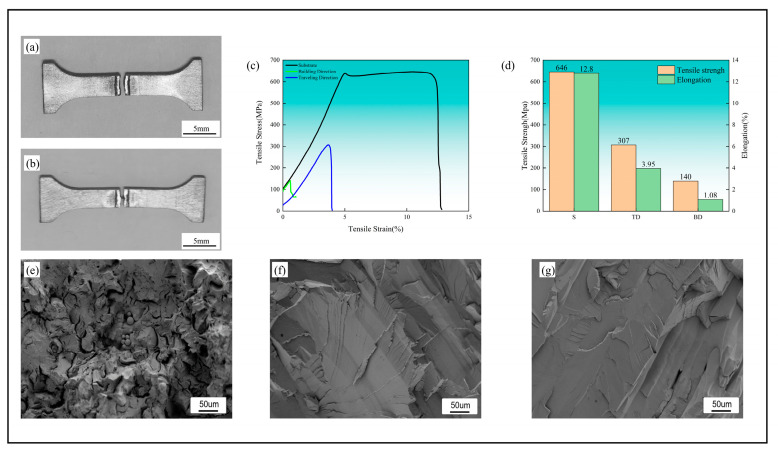
Tensile test results and fracture morphology: (**a**) Tensile specimen of the weld in the building direction. (**b**) Tensile specimen of the weld in the traveling direction. (**c**) Stress–strain curve. (**d**) Bar chart of tensile strength and elongation. (**e**) Fracture morphology of the base material. (**f**) Fracture morphology of the weld in the building direction. (**g**) Fracture morphology of the weld in the traveling direction.

Representative tensile specimens and their corresponding fracture locations are illustrated in Figure 4a,b. Fracture occurrence is consistently localized at the specimen center, specifically within the weld joint region, with fracture propagation following the weld seam path. The macroscopic examination reveals minimal plastic deformation, with fracture surfaces exhibiting predominantly planar morphology.

The stress–strain response of the Ta10W alloy is presented in Figure 4c. The additive Ta10W base material demonstrates a tensile strength of 646 MPa with an elongation of 12.8%. The presence of longitudinal cracking within the weld zone results in compromised mechanical integrity. The traveling direction weld exhibits a tensile strength of 307 MPa, representing 48% of the base material strength, with an associated elongation of 3.95%. In contrast, the building direction weld achieves a tensile strength of 142 MPa, corresponding to 22% of the base material strength, with an elongation of 1.08%. The superior tensile strength observed in the traveling direction weld correlates with the reduced defect density compared to the building direction weld.

The SEM examination of the Ta10W alloy fracture morphology is presented from Figure 4e–g. The base material exhibits mixed-mode fracture characteristics, with observable unmelted spherical powder particles on fracture surfaces. Welds in both directional orientations demonstrate brittle cleavage fracture mechanisms, characterized by distinctive river pattern morphology on fracture surfaces. 

The enhanced mechanical properties associated with fine-grained structures can be attributed to increased grain boundary area and pronounced grain orientation differences, which effectively impede dislocation motion, resulting in improved strength and toughness characteristics. Furthermore, fine-grained structures facilitate uniform deformation distribution, mitigating localized stress concentration and suppressing solidification crack formation, thereby enhancing plasticity and fracture toughness. Conversely, the coarse-grained structure observed in the building direction, characterized by reduced grain boundary density and lower dislocation mobility constraints, may promote stress concentration and crack propagation, particularly in {001} textured regions which exhibit limited slip systems and increased cleavage fracture susceptibility. Consequently, the {101} texture and fine-grained characteristics in the traveling direction demonstrate enhanced resistance to solidification cracking, while the {111} and {001} textures in the building direction exhibit increased fracture sensitivity and reduced plasticity.

Both constituent elements of the Ta10W alloy—tantalum and tungsten—crystallize in body-centered cubic (BCC) structures, forming a solid solution characterized by tungsten dissolution in the tantalum matrix. Cleavage fracture mechanisms predominantly occur in alloys exhibiting hexagonal close-packed (HCP) and BCC structures, typically manifesting brittle fracture behavior. Cleavage fracture propagation occurs along specific crystallographic planes, designated as cleavage planes. In polycrystalline materials, grain boundaries function as barriers to cleavage crack propagation. Dislocation pile-up at grain boundaries generates localized stress concentration phenomena. The accumulated elastic energy associated with increasing dislocation density at grain boundaries may exceed the surface energy increment required for cleavage crack propagation along corresponding planes in adjacent grains, initiating multi-directional cleavage crack formation at grain boundary intersections. This sequential process manifests throughout the material volume, resulting in cleavage crack formation and ultimate material failure, characterized by the observed river pattern morphology on fracture surfaces.

## 4. Conclusions

The laser welding of SLM-fabricated Ta10W alloy demonstrates performance degradation in both traveling and building directions, with significant longitudinal crack formation observed within the weld zones. Specifically, when welding in the traveling direction, the grain size within the weld decreased by 25.1%. However, the tensile strength and elongation of the weld seam were only 48% and 30.9% of that of the base material, respectively. In contrast, when welding in the building direction, the average grain size of the weld increased by 64.8%, with the tensile strength and elongation reaching only 22% and 8.4% of the base material’s values.

These experimental findings indicate that optimization of welding parameters, particularly welding direction selection (preferentially the traveling direction) and heat input control, can significantly enhance weld performance and minimize defect formation, establishing a viable technical approach for reliable large-scale structural component fabrication.

In aerospace applications, this methodology demonstrates particular relevance for high-temperature structural component fabrication, specifically in rocket engine nozzle manufacturing. The established relationships between welding direction, grain refinement, and mechanical properties provide fundamental insights for enhancing the high-temperature performance and fatigue resistance of critical components. The characterized weld fracture mechanisms indicate that optimization of laser welding process parameters effectively suppresses solidification crack formation and porosity defects, thereby enhancing weld integrity. This precision manufacturing technology aligns with aerospace industry requirements for complex geometric configurations and extreme operating conditions.

## Data Availability

The original contributions presented in the study are included in the article, further inquiries can be directed to the corresponding author.
